# Severe Neonatal Presentation of Cornelia de Lange Syndrome With Fatal Outcome: A Case Report

**DOI:** 10.7759/cureus.108947

**Published:** 2026-05-16

**Authors:** Chaymae Cherrabi, Anass Ayyad, Mohammed Ech-Chebab, Sahar Messaoudi, Rim Amrani

**Affiliations:** 1 Department of Pediatrics, Centre Hospitalier Universitaire Mohammed VI, Oujda, MAR; 2 Department of Neonatology, Faculty of Medicine and Pharmacy, Mohammed First University, Oujda, MAR; 3 Department of Neonatology, Centre Hospitalier Universitaire Mohammed VI, Oujda, MAR

**Keywords:** cornelia de lange syndrome, fatal outcome, neonatal presentation, respiratory distress, shock

## Abstract

Cornelia de Lange syndrome (CdLS) is a rare genetic disorder characterized by a wide spectrum of clinical severity, ranging from mild to severe forms, and is typically associated with distinctive facial features, growth retardation, and multiple congenital anomalies. We report the case of a 10-day-old male newborn, born at term to non-consanguineous parents, who was admitted for respiratory distress, feeding refusal, and hypotonia. On examination, the patient presented with shock, cyanosis, severe respiratory distress, and marked hypotonia. Dysmorphic facial features were noted, along with bilateral ectrodactyly of the hands, micropenis, and bilateral cryptorchidism. Biological and radiological investigations did not identify any infectious or structural etiology. The diagnosis of CdLS was established based on clinical findings. Despite intensive care management, the patient's condition rapidly worsened, culminating in cardiac arrest with unsuccessful resuscitation. This case highlights a severe neonatal presentation of CdLS with a fatal outcome and underscores the importance of early recognition, multidisciplinary management, and genetic counseling, given the poor prognosis associated with severe forms.

## Introduction

Cornelia de Lange syndrome (CdLS) is a rare genetic syndrome, with a prevalence rate of about one in 40,000 births. It is considered one of the complex congenital syndromes [1]. The syndrome displays a wide spectrum of clinical manifestations, varying from mild to severe types.

Clinical features of CdLS include typical facial dysmorphia, growth failure before and after birth, excessive hairiness, and problems with the upper limbs, which could be either minor phalangeal abnormalities or even severe oligodactyly [2]. Other systems can be affected, especially the heart, digestive system, and lungs.

From the perspective of genetics, the syndrome is heterogenic, characterized by mutations that arise within cohesin genes. The most important discovery was the detection of the Nipped-B-like protein (NIPBL) gene, after which a number of other genes, including structural maintenance of chromosomes 1A (SMC1A), were discovered, which highlighted the significance of this complex for gene expression and chromosome stability [3].

Expressivity in relation to the condition seems fairly consistent among family members. Although individuals will not necessarily have their life span shortened in case there is no severe internal organ involvement, the occurrence of serious congenital defects would mean a bad prognosis and could lead to early death. Quality of life will also be affected due to cognitive delays, physical defects, as well as behavior and psychiatric problems [3,4].

Diagnosis is primarily based on clinical findings, supported by genetic analysis. Management is multidisciplinary and aims to improve both prognosis and patients' quality of life.

This case is of particular interest because of the variety of clinical manifestations observed and the characteristic dysmorphic features suggestive of CdLS, which helped guide the clinical diagnosis. It also highlights the importance of the international consensus criteria in the diagnostic evaluation, particularly in settings where genetic testing is not readily available. Finally, this case emphasizes the value of early recognition of this rare condition in order to ensure appropriate management and multidisciplinary follow-up.

## Case presentation

The patient is a 10-day-old baby boy of non-consanguineous parents, second among two siblings, born by term delivery in the hospital. There was no family history of similar cases, and the parents were not of advanced age. The infant has been admitted to our center because of breathing difficulty and inability to feed and with a history of muscle hypotonia starting a day before admission.

**Figure 1:**
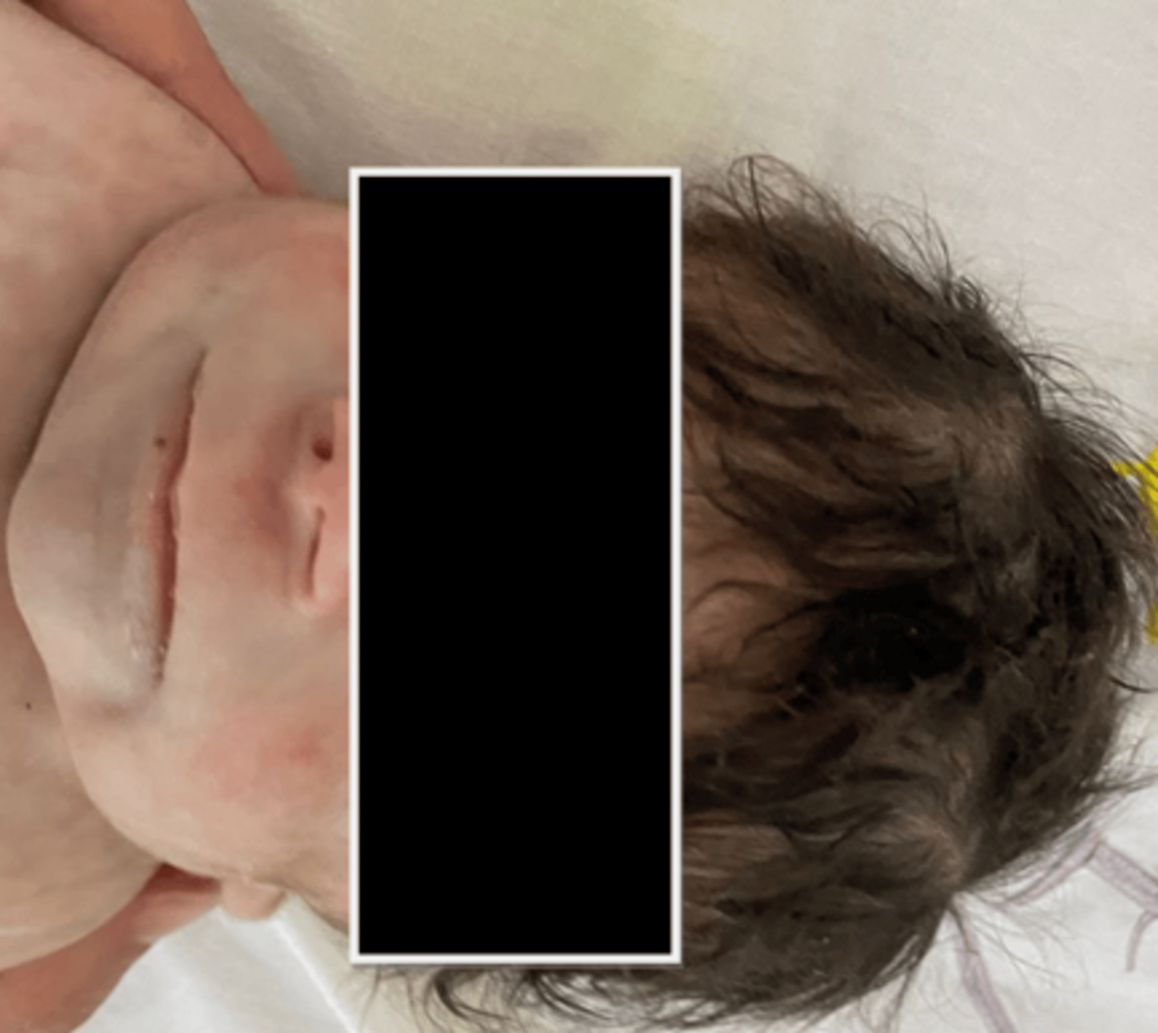
Facial dysmorphism Facial dysmorphism with synophrys, thick eyebrows, short nose with an upturned tip, long philtrum, and thin lips with downturned corners of the mouth.

**Figure 2:**
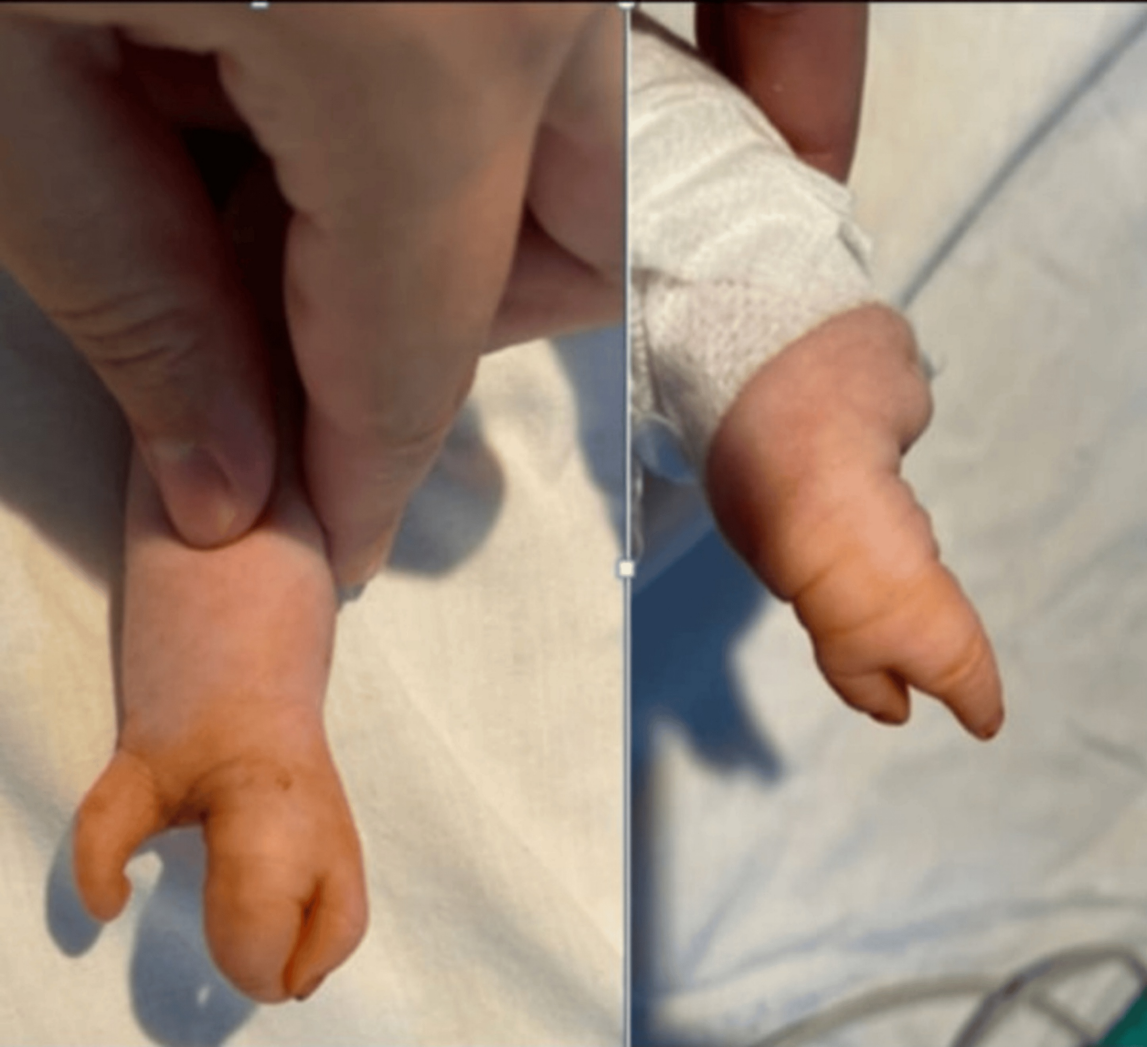
Ectrodactyly Ectrodactyly of both hands.

Clinical examination upon admission showed that the baby was in a state of shock, cyanotic, mottled, hypotonic, and hyporesponsive with a heart rate of 190 beats/min, severe respiratory difficulty with a Silverman score of 4/10, and a symmetrical severe intrauterine growth retardation. Regarding dysmorphia, clinical examination showed facial dysmorphism (Figure 1), associated with bilateral ectrodactyly of the hands (Figure 2) along with a micropenis with bilateral cryptorchidism.

In the initial laboratory tests, the patient had a total white blood cell (WBC) count of 15,010/mm³, where there were 8,570/mm³ neutrophils and 5,050/mm³ lymphocytes, a hemoglobin (Hb) level of 13 g/dL, and a platelet count of 202,000/mm³. The test for C-reactive protein came out negative, whereas the procalcitonin was positive. Electrolyte levels in serum were normal. There was a slight decrease in renal function. Tests for cerebrospinal fluid analysis and urine culture were negative. Bacteria cultured from blood included *Staphylococcus hominis*, which was thought to be contamination. Chest radiography showed that the heart had a normal size without any signs of cardiomegaly, but with a bilateral interstitial pattern and no evidence of parenchymal involvement. Transthoracic echocardiogram showed no cardiac anomalies.

Abdominal and genitourinary ultrasounds were normal and showed no additional abnormalities. A constitutional karyotype was conducted and yielded normal results with no chromosomal abnormalities identified. In light of the presence of facial dysmorphism, ectrodactyly, and growth delay, a diagnosis of CdLS was made. This diagnosis was supported by the international consensus scoring system, with a total score of 14 points (Table 1).

**Table 1:** International consensus diagnostic criteria and scoring system for Cornelia de Lange syndrome in the present case The diagnosis of Cornelia de Lange syndrome was evaluated using the international consensus scoring system [1], based on cardinal and suggestive clinical criteria. The total score was calculated according to the presence or absence of each feature in the patient. In the present case, the total score was >11, consistent with a classical form of Cornelia de Lange syndrome.

Diagnostic criteria of Cornelia de Lange syndrome	Findings in the patient	Score
Cardinal criteria (2 points/criteria)		
Synophrys and/or thick eyebrows	Yes	2
Short nose with upturned nasal tip	Yes	2
Long and/or smooth philtrum	Yes	2
Thin upper lip with downturned corners	Yes	2
Upper limb reduction defects	Yes	2
Congenital diaphragmatic hernia	No	0
Suggestive criteria (1 points/criteria)		
Pre-/postnatal growth retardation	Yes	1
Microcephaly	Yes	1
Psychomotor/intellectual developmental delay	Yes	0
Hirsutism	Yes	1
Small hands and/or feet	Yes	1
Hearing loss	No	0
Total score		14 points

The disease had a progressive course leading to respiratory failure requiring sedation and intubation with mechanical ventilation. Despite the best intensive care management, the child experienced cardiac arrest, from which he did not recover.

## Discussion

CdLS is a rare multi-systemic genetic disease which is defined by abnormalities in physical and mental development. The syndrome is associated with severe phenotypical heterogeneity and named after Dutch pediatrician Cornelia de Lange, who discovered this medical condition for the first time in 1933 among two babies [1,5]. The disease is rare; however, the incidence ranges from one out of 10,000 to 50,000 children born alive. There are no particular differences concerning the frequency between males and females or any racial groups, which implies its worldwide prevalence [6].

The clinical diagnosis can be established in the case of the appearance of specific clinical symptoms. Among them, a distinct face with dysmorphism can be noticed, and it includes microcephaly, synophrys (arched and thick eyebrows), long eyelashes, a high-arched palate, a short nose with the upturned tip, a long or smooth philtrum, and thin upper lips. Besides, patients suffer from dental anomalies, widely spaced and small teeth.

Growth retardation that occurs during the prenatal stage and even after birth is commonly noted. This is linked to delays in psychomotor development and mental retardation of varying degrees. Limb deformities can also be seen, which vary from the severe ones where there is considerable absence of upper extremities to those with oligodactyly, clinodactyly of the fifth finger, and small hand size. Another symptom that is frequently mentioned is hypertrichosis, in which excess hair formation occurs on the face, ears, back, and limbs. CdLS is one that involves various systems in the body and is marked by congenital defects [2].

Heart anomalies are found in around 20-30% of the affected individuals, commonly presenting with atrial and ventricular septal defects. Digestive tract anomalies are common, mainly characterized by gastroesophageal reflux found in over 70% of cases and occasionally in serious forms, besides intestinal anomalies. Respiratory problems manifest as a higher susceptibility to recurrent respiratory tract infection and ventilator problems. Genitourinary system anomalies are also common, mostly seen in males, and mainly involving cryptorchidism in about 60-80% of patients. ENT problems also exist in about 40% of the cases, namely, conductive and sensorineural hearing impairment [2]. In our case, several clinical features supported the diagnosis. The newborn presented with severe growth retardation, associated with suggestive facial dysmorphism. Limb involvement was particularly marked by bilateral ectrodactyly, consistent with the spectrum of limb anomalies described in CdLS.

Genetically, CdLS is a complex disease characterized by alterations in genes encoding components of the cohesin protein complex. The identification of the NIPBL gene, mapped to chromosome 5, was a milestone in the elucidation of the molecular mechanism of the disease. This gene is important for the cohesion of chromosomes and modulation of gene expression. Additional genes contributing to this complex, such as SMC1A, were identified, and the inclusion of CdLS in the category of cohesinopathies was confirmed [7].

In this context, a clinical scoring system has been suggested to help recognize CdLS by using the correlation between major clinical criteria, such as distinctive facial dysmorphisms, growth delay, upper limb abnormalities, and hypertrichosis, and minor criteria, such as genitourinary malformations and multiple organ dysfunction [1]. The presence of these symptoms makes it possible to differentiate between classic and less severe cases. In the current case, genetic confirmation could not be done at an earlier stage; therefore, the diagnosis was made clinically [1].

Management of CdLS is supposed to be multifaceted and ongoing, involving frequent medical assessment, genetic counseling, and screening for complications that may occur. This will enhance the quality of life of the patients [1].

The prognosis for CdLS is determined by the intensity of symptoms. In the most severe cases, it may result in premature death, primarily during infancy, because of issues related to breathing, infections, and serious congenital abnormalities. Problems with eating and breathing can lead to aspiration pneumonia, which worsens the prognosis significantly. On the other hand, less severe cases may result in individuals living up to adulthood, with outcomes varying according to the extent of brain dysfunction and other problems [8]. However, in severe cases, such as ours, the prognosis may be highly unfavorable, especially within the neonatal period [8].

## Conclusions

CdLS is a very rare condition and shows great clinical variability; features include growth delay, dysmorphic facies, and multiple congenital abnormalities. The current case describes an extremely severe presentation in a neonate with a rapid death despite optimal treatment. The case reinforces the necessity of recognizing severe cases of CdLS at an early stage because of the potential rapidity with which lethal complications can develop.
